# Adjustment of K^+^ Fluxes and Grapevine Defense in the Face of Climate Change

**DOI:** 10.3390/ijms221910398

**Published:** 2021-09-27

**Authors:** Houssein Monder, Morgan Maillard, Isabelle Chérel, Sabine Dagmar Zimmermann, Nadine Paris, Teresa Cuéllar, Isabelle Gaillard

**Affiliations:** 1 BPMP, Univ Montpellier, CNRS, INRAE, Institut Agro, F-34060 Montpellier, France; houssein.monder@inrae.fr (H.M.); morgan.maillard@inrae.fr (M.M.); isabelle.cherel@inrae.fr (I.C.); sabine.zimmermann@cnrs.fr (S.D.Z.); nadine.paris@cnrs.fr (N.P.); 2 CIRAD, UMR AGAP, Univ Montpellier, INRAE, Institut Agro, F-34398 Montpellier, France; teresa.cuellar@cirad.fr

**Keywords:** potassium homeostasis, potassium transport, fruit, quality, climate change

## Abstract

Grapevine is one of the most economically important fruit crops due to the high value of its fruit and its importance in winemaking. The current decrease in grape berry quality and production can be seen as the consequence of various abiotic constraints imposed by climate changes. Specifically, produced wines have become too sweet, with a stronger impression of alcohol and fewer aromatic qualities. Potassium is known to play a major role in grapevine growth, as well as grape composition and wine quality. Importantly, potassium ions (K^+^) are involved in the initiation and maintenance of the berry loading process during ripening. Moreover, K^+^ has also been implicated in various defense mechanisms against abiotic stress. The first part of this review discusses the main negative consequences of the current climate, how they disturb the quality of grape berries at harvest and thus ultimately compromise the potential to obtain a great wine. In the second part, the essential electrical and osmotic functions of K^+^, which are intimately dependent on K^+^ transport systems, membrane energization, and cell K^+^ homeostasis, are presented. This knowledge will help to select crops that are better adapted to adverse environmental conditions.

## 1. Introduction

In plants, potassium (K^+^) is the most abundant inorganic cation in plant cells. In the cytosol, the K^+^ concentration is found in the 80–100 mM range [1,2], and the control of the cytosolic K^+^ homeostasis is essential to keep the cell alive [3,4,5]. This ion is involved in a plethora of metabolic, physiological, and developmental processes. K^+^ is a highly mobile osmolyte and a major component of the cation/anion balance. K^+^ is also crucial for the control of the membrane polarization and the neutralization of organic acids and anionic groups [6,7,8]. It contributes to cell turgor maintenance, cell elongation, or expansion, to mechanical processes such as stomatal opening/closure or leaf movements, and to the control and maintenance of cytosolic pH homeostasis. High K^+^ concentrations are required for optimal protein synthesis, activation of more than 70 enzymes, and photosynthesis [6,9]. Moreover, K^+^ is involved in the plant responses to biotic and abiotic constraints inducing quite often a K^+^ loss from the cell [3,10,11,12]. More generally, K^+^ starvation is known to affect crop yield and the use of potassium fertilizers is a vital input in agriculture aiming to maintain optimal levels of nutrient.

Many studies indicate that the universal functions of potassium are dependent on the K^+^ translocation allowing its distribution in all plant tissues and subcellular compartments of cells [4,5]. In plants, K^+^ translocation processes are achieved by the fine control of a specialized network of K^+^ transport systems, whose members can differ in transport affinity, energetic coupling, voltage-sensitivity or ionic selectivity [7,8,13]. Among the many plant K^+^ transporters and channels, Shaker-like voltage-dependent K^+^ channels have been extensively studied [7,8,14,15,16]. These ion channels allow large passive fluxes of K^+^ that dominate the K^+^ conductance at the cell plasma membrane and are implicated in the control of long-distance K^+^ transport [7,8], Mechanistically, such voltage-gated K^+^ channels mediate the inwardly or outwardly rectifying K^+^ fluxes of the plasma membrane according to the electro-chemical gradient, thus allowing the inward or outward K^+^ translocation of the cell [7,8]. They are involved in the fine-tuning of K^+^ fluxes, which adjust the cytosolic K^+^ homeostasis necessary to maintain cell viability.

However, nowadays, persistent climate changes and abrupt fluctuations in climatic conditions are imposing greater environmental constraints, with increasing temperatures, multiple droughts, and intense solar radiation. All these abiotic stressors represent serious threats to plants, impacting plant growth and development worldwide. Among other effects, abiotic stress disturbs K^+^ translocation and homeostasis, modifying the activities of K^+^ Shaker channels either by inducing transcriptional changes or modifying post-translational regulatory mechanisms [17]. In grapevine (*Vitis vinifera* L.), which is one of the most economically important fruit crops, climate change factors disturb the synthesis and degradation of primary and secondary metabolites directly, via the regulation of their biosynthetic pathways, or indirectly, via their effects on vine physiology and phenology [18,19,20,21]. In particular, grape harvest occurs nowadays on average three weeks earlier in the summer as compared to the situation 25 years ago [22,23]. This means that the entire period of berry ripening has been shifted toward warmer periods. Moreover, plants must endure more frequent heat waves associated with excessive solar radiation and soil dehydration, which leads to plant transpiration and water stress [14,24,25,26]. The current climate constraints directly increase K^+^ loading in the berry. This excess K^+^ during ripening leads to a decrease in grape acidity at harvest [14,15,27,28]. In addition, an increase of sugar loading in the berry is observed [29]. This disturbs the grape berry composition and has a strong impact on the final quality of the obtained wine. Thus, adaptation to climate changes is becoming a major challenge for viticulture. 

This review deals with the fine control of K^+^ fluxes at the plasma membrane of grape berry cells in relation with climate changes. Because this control is strongly dependent on K^+^ transport systems and that the Shaker-like voltage-dependent K^+^ channels have been intensively studied, their involvement is particularly discussed. After a short description of grape berry development and its requirement for K^+^, the first part of this review discusses the detrimental climate constraints on grapes during their development and ripening which in turn disturb the production of wine. The second part focuses on the role of K^+^ in the initiation and the maintenance of berry loading during ripening, and its involvement in the defense mechanisms against abiotic stresses in relation to climate change.

## 2. Grape Berry and Climate Change

### 2.1. The Development of Grape Berries

The development of grape berries consists of two successive growth periods separated by a lag phase. The first period comprises a rapid cell division phase followed by marked cell enlargement, and is characterized by the synthesis and vacuolar storage of the two main grape organic acids, tartaric acid and malic acid. At the onset of ripening, the berry starts to change color and soften, and the fruit undergoes major biochemical changes [30]. From this step, berry growth is only due to cell expansion and potassium as an osmoticum in driving cell growth and turgor, is expected to be essential for this process [15,27,30,31,32]. During ripening, grape berry, becomes a strong sink for K^+^ and this cation also drives sugar accumulation via the phloem [1,15,27,33,34]. Moreover, transport mechanisms switch from the symplastic to the apoplastic mode [35], meaning that solutes must cross plasma membranes at least twice before accumulating within the berry flesh cells. Previous reports have indicated that berry loading via an apoplastic mode should improve the control of efficient long-distance phloem transport from source leaves to ripening berries [27,35]. It is important to note that K^+^ channels are involved in the control of K^+^ and sugar translocation from source to sink via the phloem [1,36], using the transmembrane electrochemical gradient for K^+^ between phloem cytosol and berry apoplast that allows the berry loading process [33,37,38].

For the whole grape maturing period, an adequate K^+^ availibility is a prerequisite for a normal berry development. Grapevine is able to adapt to different availabilities of K^+^ via responses that mainly involve changes in root architecture and regulation of K^+^ transport systems. This plant also invests energy for the uptake of K^+^ and its distribution throughout the plant. Indeed, although the K^+^ concentrations of the soil solution are generally very low, highly variable, and usually in the range of 1 to 0.1 mM [39], grapevine can accumulate K^+^ to maintain high cytosolic concentrations at around 100 mM [40,41]. Viticultural practices attempt to mitigate the variability in K^+^ availability using fertilizers to try to maintain an optimal K^+^ level. But, with regard to vines cultivated on sandy soils located on the sea front, the occurrence of additional salt stress disturbs K^+^ nutrition causing deficiency [42,43]. Drought stressed grapevine may also need additional K^+^. It is widely known that potassium fertilization improves water relations and osmotic adjustment and the response of vine are often variety dependent [43]. Tightly linked, it is important to note that K^+^ starvation reduces also the ability to use N and induces chlorosis at the tip of older leaves disturbing plant metabolism [44]. 

### 2.2. The Impact of Climate Change on Grapevine

Due to the greenhouse effect, higher atmospheric CO_2_ concentrations have led to increased global temperatures associated with episodes of poor water availability and drought. In addition, the incidence, intensity, and duration of extreme heat waves have increased [45,46]. Grapevine is a perennial crop known for its ability to survive long periods of drought and high temperature exposure. However, for optimal fruit production and quality, grapevine needs to grow in a suitable environment that provides a good range of atmospheric temperatures, appropriate radiation intensities and duration, and water availability [47]. Among the climate parameters that mostly affect berry content at harvest, temperature and water availability play a prominent role.

In grapevine, heat stress causes permanent injuries that affect its phenology and grape yield. Heat stress also promotes vegetative growth, disturbing flower set, and the development of young berries [48]. In addition, phloem transport is increased and berry primary and secondary metabolisms are modified [30]. This results in higher accumulation of sugars and K^+^ during fruit ripening, and leads to wines whose ethanol content increases steadily over the vintages, which is associated with reduced acidity because over-accumulated K^+^ ion leads to an excessive neutralization of organic acids. This loss of acidity strongly affects wine quality since acidity normally allows the flavor and the aroma to develop during vinification. Upon high-temperature exposure, various damages that disturb the plant’s carbon balance and cellular homeostasis also occur, potentially leading to a severe delay in growth and development [49]. The maintenance of cellular homeostasis is essential for the resilience of plant life to adverse environments. In particular, the control of K^+^ homeostasis is crucial for maintaining the many physiological processes in which K^+^ is involved [4,5,50].

In addition to an increase in atmospheric temperature, a warmer climate leads to more solar exposure for grape berries, which can be detrimental to grape quality. However, the classical viticultural practice of removing leaves in the fruit zone is known to control the source-sink balance and improves grape composition and the resulting wines [51,52]. When exposed to sunlight that contains a fraction of emitted radiation in the UV spectrum (110–400 nm), grapevine plants activate enzymes and secondary metabolites that belong to antioxidants and defense-related phytochemicals. Indeed, the observed changes in berry composition improve grape berry quality at harvest and explain why grapevine is generally considered to be well-adapted to UV radiation [53,54,55,56]. However, this cultural defoliation practice is under question since the infrared portion of sunlight radiation is also increased and transmits thermal energy to grape berries, resulting in an intrinsic increase in their temperature. This strongly desynchronizes the metabolism of sugar and organic acids and decreases anthocyanin content, disturbing fruit quality [57,58].

The water requirements of plants increase in warmer climates since higher temperatures promote plant transpiration and contribute to soil dehydration, resulting in the occurrence of longer and more frequent water stresses. Water restriction has major effects, which are not always detrimental, on grape production and composition. The impact of water stress on grapevine depends on its intensity, duration, and occurrence within the development cycle [59]. Indeed, a moderate water stress that occurs near the start of ripening or at later development stages in berries improves sugar and K^+^ content, flavonoid compounds, and the organoleptic quality of the produced wines [59].

In contrast, even moderate water stress close to bloom time leads to poor fruit setting and the abortion of the entire clusters. Stress applied during the short initial berry cell division phase results in the significant reduction of berry size and quality [59]. Indeed, an early water deficit can have detrimentally irreversible effects associated with stomatal closure, due to abscisic acid (ABA) long-distance signaling produced by dehydrating roots [60]. This further induces a decrease in CO_2_ uptake and the downregulation of photosynthetic capacity and growth. It must be noted that the stomata are key players in the plant response to drought, and they close in order to reduce transpiration and avoid water loss. In grapevine, the different varieties exhibit various sensitivities to water deficit, meaning that some of them tend to close their stomata earlier than others [61,62,63].

Abundant documentation exists on the description of vine responses to abiotic stresses, which involves adaptive changes and/or deleterious effects to grape quality [26,46,56,63,64,65]. Under field conditions and in the context of climate change, these responses could be modified by the simultaneous combination of different climatic stresses. Until now, most existing data have been restricted to the impact of a unique stress on grape development and quality. However, recent reports indicate that plant responses to combined stresses are not the summation of the plant responses to each isolated stress; instead, various types of antagonistic, synergistic, and null responses can occur [66,67]. Gene regulatory networks that control individual and multiple stress responses can be different. Generally, it is reported that combined stresses lead to the contribution of more integrative networks in the model plant *Arabidopsis thaliana* [68] and other plants including grapevine [69,70,71]. Indeed, in tomato plants, under the combination of heat and salt stress, a high K^+^ concentration is observed leading to a lower Na^+^/K^+^ ratio, a better performance of cell water status and photosynthesis as compared to salinity alone [69]. Moreover, when K^+^ is added to watering, a better performance of the antioxydant enzymes and photosynthesis parameters are noted for this stress [70].

In the plant model *A. thaliana*, an extensive study on the impact of multi factorial stress, including heat and high light stress combination, shows that the expression of genes belonging to the ABA signaling are significantly regulated [72]. The phytohormone ABA plays an essential role in responses to abiotic stresses [73,74] operating at the whole plant level and regulating processes such as leaf transpiration and water loss through stomatal closure. ABA is translocated to target tissues in both xylem and phloem, allowing transport in both direction between roots and shoots [75]. At the end of the ABA pathway, coordinated K^+^ and anion fluxes across cell membranes occur, mediated by different transport systems. Zandalinas et al. showed in their RNA-seq analyses that genes coding for targets of environmental stresses, including Shaker-like K^+^ channels and their molecular regulators, are significantly up- or down-regulated in combined stress conditions [72]. In grapevine, salt and drought stress responses seem to be regulated by the stress phytohormone ABA [76]. Moreover, a recent study analyzed the responses of two contrasted vine varieties under drought, heat stress, and high light either individually or in a combination of two or three stresses. These results are in accordance with an involvement of ABA pathways and revealed contrasted responses between the two studied varieties [71].

To cope with solar radiation, warm temperature, and water stresses imposed by the current climate, plants need to sense nutrient levels in the surrounding organs and tissues and inside their cells. Then, they must accordingly adjust various transport steps leading to nutrient accumulation into the cells with respect to their homeostasis range. Maintenance of high cytosolic K^+^ is achieved by the precise adjustment of K^+^ fluxes that are controlled by K^+^ transport systems expressed in the different membranes. Shaker-like voltage-dependent K^+^ channels allow large passive fluxes at the cell plasma membrane and have been therefore intensively studied.

## 3. Structure and Function of Plant K^+^ Shaker Channels

Plant voltage-gated K^+^ channels are referred to as “plant Shakers” in reference to animal Shaker channels, which were named for a phenotype of jerky movements and shaking observed in fruit flies deficient in K^+^-channel activity [77]. Although they belong to the same superfamily, it is now recognized that plant voltage-gated K^+^ channels and metazoan Shaker channels are derived from distinct prokaryotic ancestors [78,79]. Plant voltage-gated K^+^ channels, which are localized in the plasma membrane of expressing cells, play crucial roles in sustained K^+^ transport [7,8,14,80]. Functional channels are tetramers composed of four often non-identical α-subunits. *In planta*, the preferential assembly of these subunits as heterotetrameric structures increases channel functional diversity since the newly obtained channels acquire functional properties that differ from those of homotetrameric channels [81,82,83]. Shaker subunits have an identical structure and contain six membrane-spanning domains (S1–S6) and a long hydrophilic C-terminal cytosolic region in which several domains have been identified. The first domain, named C-linker (around 80 amino acids in length) [84,85] is followed by a cyclic nucleotide-binding domain (CNBD), and then by an ankyrin domain (involved in protein–protein interaction; [86,87]), and finally by a domain named K_HA_ that is rich in hydrophobic and acidic residues and involved in channel clustering [88]. At the level of the transmembrane region, there is a highly conserved domain named P (pore), located between S5 and S6, comprising a GYGD motif, which is the hallmark of highly K^+^-selective channels. In addition, a voltage sensor is found within the S1-S4 region with the S4 transmembrane segment containing important positive charges [7,8].

**Figure 1:**
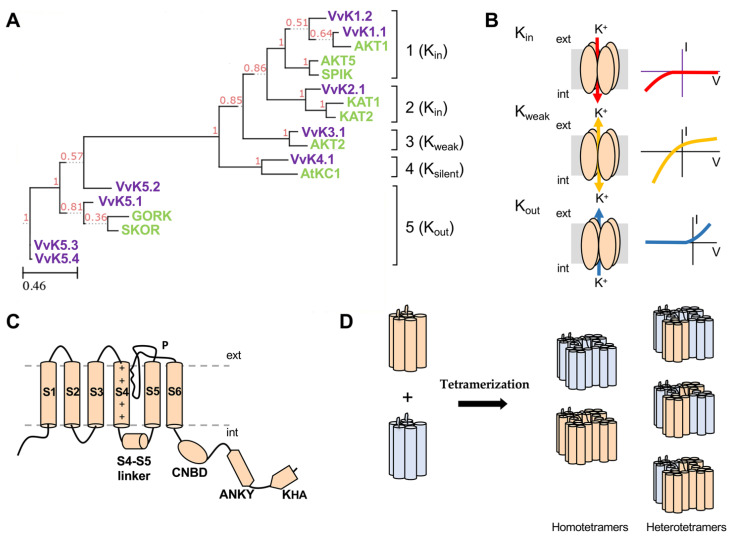
The Shaker K^+^ channel family. (**A**) Phylogenetic relationships in the grapevine (in green) and *A. thaliana* (in blue) Shaker K^+^ channel families. The Shaker family displays five groups in plants [115] named 1 to 5. Accession references are listed in Table 1. The abbreviations K_in_, K_out_ K_weak_, and K_silent_ are explained in the legend of Table 1. To find the conserved region, *A. thaliana* and grapevine Shaker polypeptide sequences were first aligned using MUSCLE 3.8.31 in full mode and then treated with Gblocks for alignment curation. The phylogenetic analyses were carried out using maximum likelihood with Phy ML 3.1/3.0 aLRT software. Tree rendering was performed using the tree drawing engine *ETE 3* [116]). Bootstrap values are indicated at the corresponding nodes. The scale bar corresponds to a distance of 4,6 changes per 100 amino acid positions. (**B**) Functional Shaker channels are multimeric proteins formed by the assembly of four Shaker subunits. Current–voltage (I–V) curves illustrate the functional types found in the homotetrameric Shaker channels that form inwardly rectifying, weakly inwardly rectifying, or outwardly rectifying conductances. Int and ext: internal and external face of the plasma membrane. (**C**) Structural domains of a Shaker channel subunit. S1 to S6: transmembrane segments, CNBD: cyclic nucleotide-binding domain, ANKY: ankyrin domain (involved in protein-protein interactions, not found in all Shaker subunits), KHA: hydrophic and acidic domain. (**D**) Assembly of four Shaker alpha-subunit is a prerequisite for channel functioning. Three-dimensional representation of S1–S6 segments in a single subunit (left) or Shaker tetramers (right). Subunits are encoded either by the same gene (homotetrameric channel) or by different genes (heterotetrameric channel). K_in_ sub-units (Groups 1, 2, 3 and 4 in A) assemble as K_in_ channels, whereas K_out_ sub-units (Group 5) form K_out_ channels. No assembly could be detected between K_in_ and K_out_ channel subunits [117]. Stoichiometry studies have revealed the various possible combinations between the different subunits [117,118,119].

Nine Shaker subunits have been identified in the genome of *A. thaliana*, as in grapevine. Phylogenetic analyses have shown that they belong to five subfamilies, although the number of members within each subgroup is not strictly conserved between the two species (Table 1, Figure 1). Groups 1 and 2 (AKT1, AKT5, SPIK, KAT1, and KAT2 in *A. thaliana* [7] and VvK1.1, VvK1.2, and VvK2.1 in grapevine [28,32,89] comprise the inwardly rectifying channels (K_in_) that are activated by hyperpolarizing potentials and are closed when the driving force for K^+^ becomes outwardly directed. They can only mediate an inward K^+^ flux allowing the uptake of K^+^ into the cytosol. The members of group 1 and 2 differ in their cytosolic part as the KAT1-like channels (group 2) lack the ankyrin repeat domain which is present in AKT1-like channels (group 1). In *A. thaliana* and in grapevine, group 3 contains only one subunit. These subunits form a weakly inwardly rectifying potassium channel (K_weak_) at the plasma membrane. Depending on their phosphorylation status, these channels named AKT2 in Arabidopsis and VvK3.1 in grapevine can exist in two different gating modes: a time-dependent voltage-activated mode mediating K^+^ influx (K_in_) into the cell (mode 1), or a non-rectifying mode with a channel locked in the open state in its phosphorylated form (mode 2) across the entire physiological voltage range allowing influx and efflux dependent on the K^+^ electrochemical gradient [90]. These two gating modes allow K_weak_ channels to mediate both, K^+^ efflux and K^+^ influx, and so they can function in both phloem K^+^ loading in source tissues and phloem K^+^ unloading in sinks [1,33,37,38]. Group 4 contains the “silent” subunits (K_silent_; AtKC1 in *A. thaliana* [91] and VvK4.1 in grapevine), which are nonfunctional when expressed alone in heterologous functional studies but can form functional heterotetrameric channels with K_in_ and K_weak_ subunits, thus modulating their activity. 

Finally, group 5 contains the outwardly rectifying channels (K_out_), which includes SKOR and GORK in *A. thaliana* [102,104] and VvK5.1, VvK5.2, VvK5.3, and VvK5.4 in grapevine [103]. These channels are activated under membrane depolarization and are closed when the driving force for potassium is inwardly directed. These K_out_ channels mediate only outward K^+^ fluxes from the cytosol to the apoplast. 

In *A. thaliana*, the role of these channels has been investigated *in planta*. The obtained results indicate that the K_in_ channel AKT1 is involved in K^+^ uptake from the soil, preferentially in a heterotetramer structure together with the regulatory subunit AtKC1 [99,105]. K^+^ secretion in the xylem sap is driven by the K_out_ channel SKOR [102]. Finally, AKT2, a K_weak_ channel that allows K^+^ influx and efflux, is involved in K^+^ (re)circulation, by controlling the phloem K^+^ loading/unloading, and in regulation of the cell membrane polarization [36,37,97]. In the extensively studied model of guard cells, K^+^ fluxes responsible for stomatal movements are mediated by the K_in_ channels KAT1 and KAT2 and the K_out_ channel GORK [82,93,104,106,107].

In grapevine, since excess K^+^ levels in berries may have a negative impact on wine quality, the molecular determinants of K^+^ transport are currently under investigation. The grapevine VvK3.1 channel is a weakly rectifying channel (K_weak_) that mediates both inward and outward K^+^ currents and is highly expressed in the phloem [33]. Starting from the onset of ripening, VvK3.1 is involved in the massive K^+^ efflux from the phloem cell cytosol to the berry apoplast. Switching to its non-rectifying mode, VvK3.1 drives the K^+^ efflux that allows K^+^ ions to move down their transmembrane concentration gradient (100 mM in the phloem cell cytoplasm and 0.1–1 mM in the apoplast; [1,108]). This is a major concern for grapevine, as the fruit is energetically limited due to stomata disappearance after the onset of ripening. As of this stage, when photosynthesis is stopped, ATP synthesis mainly depends on cellular respiration with malic acid as the substrate [109], which explains why K^+^ secretion into berry apoplast is dominated by “passive” K^+^ channels.

**Figure 2:**
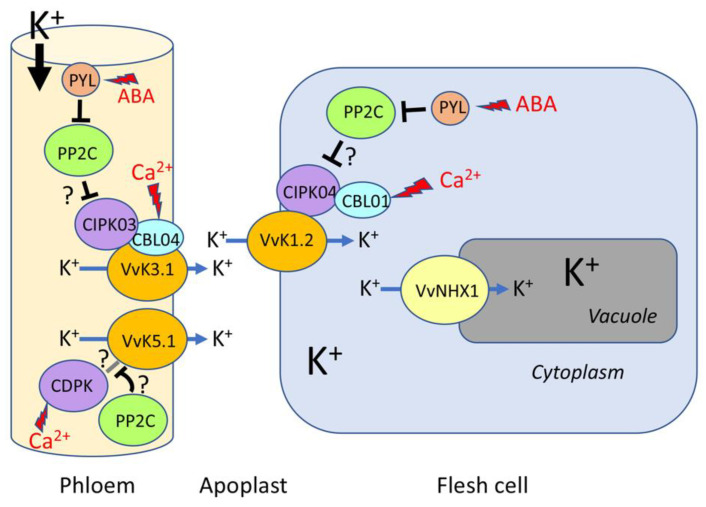
Schematic representation of known and expected K^+^ transport pathways in grape berries after the onset of ripening. K^+^ is delivered to berries via the phloem [15] and must cross the phloem plasma membrane barrier before accumulating in mesocarp cells [35]. This K^+^ flux to the apoplastic space involves the Shaker VvK3.1 channel. The activity of this channel can be enhanced by CIPK/CBL couples, possibly via an unusual mechanism of CBL anchoring in the plasma membrane [33]. Moreover, the depolarization-activated VvK5.1 channel present in phloem cells could control the plasma membrane potential [103]. By analogy with its GORK counterpart, VvK5.1 is expected to be modulated by CPKs [17]. Once in the apoplast, K^+^ is taken up by the flesh cells owing to the VvK1.2 Shaker channel [32], which recruits CIPK/CBL partners for its activation. CIPKs are also known to be inhibited by their interaction with PP2Cs of the ABA signaling pathway [17]. PP2Cs are, in an ABA-dependent manner, under the negative control of PYR/PYL/RCAR receptors. The VvNHX1 H^+^/K^+^ exchanger mediates K^+^ transfer to the vacuole, where this ion is accumulated [114].

The additional energy stored in the electrochemical gradient for K^+^ can also allow sucrose retrieval in energy-limited conditions [33,38], and it can maintain sucrose levels in the phloem vessels until reaching the different sites of unloading. At the same time, the K_in_ channel VvK1.2, an AKT1-like channel (group 1) that mediates inwardly rectifying K^+^ flux, is expressed in the plasma membrane of perivascular and flesh cells. The activity of this channel is regulated by its interactions with specific VvCIPK/VvCBL pairs, and is strongly enhanced by protons at the membrane vicinity [32] (Figure 2). In addition, it drives the rapid absorption of K^+^ into these cells to maintain the apoplastic K^+^ concentration at low levels (0.1–1 mM; [33]). Thus, the phloem stream flux toward the sink should be triggered but also retained as long as the K^+^ electrochemical gradient is maintained at the phloem plasma membrane. In parallel, a third channel belonging to the family of K_out_ channels is expressed in the phloem at the berry unloading sites. This channel, named VvK5.1, is an outwardly rectifying channel that is voltage-dependent and opens upon membrane depolarization. It is unlikely that this channel is involved in phloem unloading, since the massive K^+^ flux in the berry apoplast hyperpolarizes the plasma membrane potential. This channel is K^+^ selective and dependent on extracellular K^+^ [103,110,111]). When VvK5.1 is expressed in the phloem, it behaves like the outwardly rectifying channel GORK and is involved in the control of membrane potential via the repolarization phase [103,112].

It is important to note that once loaded into grape flesh cells by the VvK1.2 channel, K^+^ is stored in the vacuole of these cells by the transporter NHX1, a member of the NHX family [113] (Figure 2) VvNHX1 is a vacuolar cation/H^+^ antiporter that mediates Na^+^/H^+^ and K^+^/H^+^-coupled exchange, with a higher affinity for K^+^ than Na^+^ [114]. VvNHX1 expression is strongly increased starting from the onset of ripening and during berry maturation, indicating that this transporter may be responsible for the vacuolar accumulation of K^+^ during ripening, driving the uptake of water that generates vacuolar expansion [114]. 

Several other transport systems have been reported in grapevine, although they do not appear to be expressed in berries at the onset of ripening or throughout the grape ripening period. The current literature can therefore be briefly summarized in the following three points. (i) The K_in_ Shaker channel VvK1.1 is an AKT1-like channel and, like its counterpart, is expressed in the root cortical cells. Similar to AKT1, VvK1.1 is involved in K^+^ absorption from the soil. In aerial parts, its expression is very low and restricted to the phloem and the pip teguments. Interestingly, upon drought stress or ABA treatment, *VvK1.1* expression is strongly increased by about ten-fold in the berries, including during the ripening period, which could suggest a role for this channel during drought episodes [28]. (ii) The K_in_ Shaker channel VvK2.1 is an inwardly rectifying channel that displays functional properties very similar to those of KAT2. Like other KAT-like channels, VvK2.1 is expressed in guard cells and is involved in stomata opening. VvK2.1 is not expressed during grape berry ripening because the berry stomata develop into lenticels at the onset of ripening and become non-functional [89]. (iii) Two KUP/KT/HAK-type potassium transporters have also been reported [31]. HAK-KUP-KT transporters are typically selective for K^+^ and some of them are crucial when facing external solutions that contain very low K^+^ concentrations (μM range). HAK5, the most studied transporter, has been shown to mediate K^+^ uptake in roots [120,121]. In grapevine, the two studied KUP transporters VvKUP1 and VvKUP2 are expressed in the berry skin during the first period of berry development and appear to be involved in compartmentation of potassium into the skin cells during this period [31].

## 4. Regulation of K^+^ Transport in Grapevine 

To maintain the K^+^ homeostasis, plants control their K^+^ transport activity via a plethora of regulators that can precisely adjust K^+^ translocation at subcellular and cellular levels [17,122]. Studies aimed at revealing the molecular determinants of these regulations have highlighted mechanisms that likely target Shaker-like K^+^ channels at both the transcriptional and posttranslational levels. In particular, expression studies have revealed that transcript levels of the K^+^ channels *AKT1*, *AKT2*, *KAT1*, *KAT2*, *SKOR*, and *GORK* in *A. thaliana* and *VvK1.1* and *VvK1.2* in grapevine are sensitive to ABA [28,32,97,98,101,102,123,124]. Moreover, the K_weak_ (*AKT2*) and K_out_ (*GORK*) channel transcripts are upregulated under heat and drought stresses [101,125,126]. Likewise, the vine K^+^ channels *VvK1.1, VvK1.2,* and *VvK3.1* are upregulated upon drought stress [28,32,33].

As end effectors of environmental signals, channel proteins also undergo post-translational regulations, mainly governed by phosphorylation/dephosphorylation mechanisms, in response to stresses induced by climate change (Figure 2).

### 4.1. Calcium-Dependent Activation by Calcineurin B-like (CBL) Proteins and CBL-Interacting Protein Kinases (CIPKs)

To date, the CIPK family is the only described family of kinases known to have an effect on the activity of grapevine Shaker channels. CIPKs are soluble protein kinases that are activated by interacting with CBL calcium sensors within network hubs that control the transport of most major ions [127]. The binding of CBL proteins to calcium allows them to bind the auto-inhibitory domain of CIPKs, thereby leading to the activation of these kinases [128]. Activated CIPKs are positive effectors of Shaker-like channels [129,130]. These interactions are associated with certain specificities, as shown for *A. thaliana* CIPK/CBL/Shaker partners, since not all CIPKs interact with a given channel, and not all CBLs interact with a given CIPK [86,130]. CBLs also directly interact with Shaker channels [33,87]. The CIPK/CBL couple (AtCIPK23 and AtCBL1), compatible with AKT1 [28,129], was able to trigger the functional expression of VvK1.1, the orthologue of AKT1, when expressed heterologously in *Xenopus laevis* oocytes. This demonstrated that the regulatory actions of CIPK/CBL complexes are conserved in the plant kingdom.

The other inwardly rectifying channel of phylogenetic group 1, VvK1.2, is less closely related to AKT1 than VvK1.1 [32], and needs to be phosphorylated by exogenous CIPKs and CBLs in order to be active in *X. laevis* oocytes. Among the 20 CIPKs and 8 CBLs within the grapevine genome, two couples have been selected and tested: VvCIPK03/VvCBL02 and VvCIPK04/VvCBL01 (close relatives of AtCIPK6/AtCBL2 and AtCIPK23/AtCBL1, respectively), of which the latter couple is more efficient at VvK1.2 activation [32]. Thus, group 1 of vine Shaker K^+^ channels are preferentially activated by the CIPK/CBL couple, which is the most similar to that found for AKT1; the activation mechanism is likely phosphorylation as in Arabidopsis [129].

The mechanism of activation by CIPKs is expected to be quite different for VvK3.1, a member of group 3 [33]. The orthologue of VvK3.1 in Arabidopsis is the AKT2 channel. Held et al. [130] reported that the CIPK/CBL couple CIPK6/CBL4 can strongly activate AKT2 in *X. laevis* oocytes. Surprisingly, the kinase catalytic domain of AtCIPK6 was not required for the activating effect, which rules out the activation by a phosphorylation/dephosphorylation mechanism. Instead, the C-terminal regulatory domain of AtCIPK6 could interact with the AtCBL4 protein, and AtCBL4 could drive the AKT2-CIPK complex to the plasma membrane via the myristoylation and palmitoylation sites in its polypeptide sequence. Similar to AKT2 (albeit to a lower extent), VvK3.1 is activated in *X. laevis* oocytes by VvCIPK03 (or VvCIPK02/05) and VvCBL04, the closest relatives of AtCIPK6 and AtCBL4 [33]. This activation could also involve the promotion of channel targeting through the myristoylation and palmitoylation sites conserved in VvCBL04.

### 4.2. Expected Regulatory Proteins Inferred from Arabidopsis

*Type 2C protein phosphatases (PP2Cs) involved in abscisic acid signaling*: ABA plays a prominent role in the initiation of berry ripening [131,132] and in maintaining the maturation process [132,133]. During ripening, the ABA level in berries was found to be enhanced by heat stress [133]. In the ABA signaling pathway, clade A PP2Cs are central elements that are directly in contact with ABA receptors of the PYrabactin Resistance/PYR1-Like/Regulatory Component of the ABA Receptor (PYR/PYL/RCAR) family, and are inhibited by these receptors in an ABA-dependent manner [134]. PP2Cs control the activity of downstream kinases and phosphatases involved in the activation of K^+^ channels [17]. In *A. thaliana*, clade A PP2Cs of the ABA signaling pathway directly regulate the activity of Shaker-like K^+^ channels [135,136] and also inhibit or counteract the effect of kinases that activate the channels (for review see [17]. Twelve clade A PP2C genes have been identified in the grapevine genome (VvPP2C57-68 [137]). At the transcriptional level, grapevine PP2C genes are expressed in berries, and some of them respond to drought, heat, cold, and ABA treatments [132,137,138,139]. Functional studies in oocytes have shown that clade A PP2Cs inhibit the activities of AtCIPK23 (activating AKT1) and AtCIPK6 (activating AKT1 and AKT2) [140], and the ABI2 PP2C dephosphorylates CIPK23 in vitro [141]. It is therefore expected that similar regulations exist in the berry for the control of K^+^ channels during the initiation and maturation of grape berries, or in response to abiotic stresses.

*SnF1-related protein kinase 2 (SnRK2) kinases:* The OST1/SnRK2E/SnRK2.6 kinase, which is directly regulated by clade A PP2Cs [142], interacts with the *A. thaliana* channel KAT1 [143] and phosphorylates a threonine residue (T306) important for the activity of this channel [144]. However, when expressed heterologously in oocytes, this kinase has no direct effect on the activity of KAT1 [145], suggesting that T306 is not directly involved in the control of channel activity by OST1. In plants, OST1 is required for the ABA-dependent negative regulation of inward channels [143]. Two-hybrid tests have found interactions between VvK2.1 [89], the grapevine orthologue of KAT1, and two members of the SnRK2 family in grapevine, VvSnRK2.1 and VvSnRK2.4 [146].

*Calcium-dependent protein kinases (CPK/CDPK):* Besides CIPKs, other calcium-dependent kinases involved in ABA and calcium signaling are known to control the activity of Shaker K^+^ channels in Arabidopsis. These CDPKs (or CPKs) have different effects (activating or inhibiting) on channel activities expressed in *X. laevis* oocytes [147,148]. Additionally, CDPKs display different sensitivities to calcium. Calcium ions directly interact with EF-hands present in the sequence of the kinase, which relieves the auto-inhibition [149]. CDPKs can be countered by clade A PP2Cs in oocytes [150]. The grapevine genome comprises 19 CDPK genes [151], including 5 that are highly expressed in berries at mid-ripening [152]. Most of them positively respond to ABA in leaves, and a few respond to heat stress [151]. Some of these CDPKs might therefore play a role in grapevine channel regulation in response to stresses associated with global warming.

## 5. K^+^ Ions Play a Major Role in Defense Mechanisms Used to Resist Climate Change Constraints

Because nutrient homeostasis is crucial for maintaining the internal, physical, and chemical conditions of living systems, the underlying mechanisms involved in this control have been a topic of investigation in the past decade. In particular, K^+^, which is the most abundant cation in plant cells and is involved in multiple functions, needs to have its cytosolic homeostasis maintained in a concentration range compatible with biochemical processes necessary to cell life. This process is crucial to the development of plants and their resilience to hostile environments. Under stressful conditions, the plant adaptation response helps to protect K^+^ homeostasis via significant changes in the activity of K^+^ transport systems in stressed tissues and cells (see above and [41,50,153,154]). One recent study using mathematical models of membrane transporter systems has provided new insight into the functional interplay between these channels/transporters [5]. This study confirms that a single K^+^ channel or transporter cannot control the K^+^ concentration of the cytosol. At least two to three differently energized transport systems (e.g. a K^+^ channel, a H^+^/K^+^ symporter, and a H^+^/K^+^ antiporter) would be needed to adjust the cytosolic K^+^ concentration in an energy-consuming process [5,155].

Nevertheless, climate change can disturb this K^+^ homeostasis. Specifically, plants respond to warmer climates by producing reactive oxygen species (ROS), which are key players in both developmental processes and stress responses [156]. ROS are highly reactive molecules that can be reduced or excited forms of O_2_, including singlet oxygen (^1^O_2_), hydrogen peroxide (H_2_O_2_), the superoxide anion (O_2_^•−^), and the hydroxyl radical (OH^•^) [157]. Additionally, ROS have a dual action in abiotic stresses that depends on their cellular concentration [158]. Whereas low levels of ROS could be involved in the stress-signaling pathway by triggering acclimation responses, ROS become extremely harmful to cellular membranes when their concentrations reach the point of toxicity, which can lead to oxidative stress and possibly cell death [159].

Like all fruits, grape berries are a source of antioxidants including anthocyanin and phenolic compounds, with numerous adequate metabolites that allow their synthesis and regeneration. These antioxidant compounds can process ROS to delay or avoid cell damage, and for signaling processes [160]. At the same time, an abiotic stress has been observed to induce K^+^ loss in stress-affected tissues [10]. Initially observed as a part of salt stress in roots [10], this K^+^ efflux from the cytosol of stress-affected cells has been confirmed for multiple stresses including anoxia, heavy metals, drought, and temperature extremes [11,161,162,163,164], making it a common sign of plant stress responses. This K^+^ efflux is activated by hydroxyl radical (OH•) [10] and leads to a decrease in K^+^ cytosolic concentration, consequently resulting in significant changes in metabolic reactions and physiological processes. In *A. thaliana*, this K^+^ efflux is mediated by the outwardly rectifying Shaker channel GORK (K_out_) [10,100]. Under normal conditions, this channel is activated by membrane depolarization and is involved in both stomatal closure [104] and phloem membrane repolarization [103,112]. However, cytosolic K^+^ homeostasis is crucial to maintain cell viability, and it is known that dramatic K^+^ loss from the cytosol activates the catabolic enzymes involved in programed cell death (PCD) [158,165,166].

In plants, the GORK channels are modulated by K^+^ sensing, voltage gating, phosphorylation, hormonal signaling, calcium, and ROS [3,162,167,168]. Recently, it was proposed that the strong cytosolic K^+^ efflux from the cytosol of stressed cells could be a switch that inhibits metabolic reactions in order to favor defense and adaptation mechanisms [3,12]. The authors explain that this switch functions to slow down or stop the energy-consuming metabolic reactions in order to redirect ATP pools for defense mechanisms [3]. One way to decipher this mechanism may be to design appropriate strategies that will decrease the negative impact of the current climate on crop plants to avoid any significant damage. This will provide an improved basis for the selection of crops adapted to our future environment.

## 6. Conclusions

The negative impacts induced by climate change are among the major causes of crop yield decrease worldwide. Many researchers are working on this issue, investigating and testing various solutions to reach more crop resilience to challenging environment. In grapevine, temperature, high solar radiations, heat waves, and drought stresses are the climatic parameters that disturb the grape composition the most at harvest with a detrimental impact on wine quality. Since, K^+^ is involved in integrated mechanisms necessary for grape development and maturation, it explains why it is so important for berry and wine quality. Starting at ripening, K^+^ is a major actor of the initiation and the maintenance of berry loading from the phloem owing to the fine tuning of K^+^ fluxes by K^+^ transport systems. This participates both to the control of membrane potential and the maintenance of the transmembrane K^+^ gradient at the phloem cells. During ripening, K^+^ as an osmolyte is involved in osmotic adjustment and osmoprotection of the flesh cells by the control of the vacuole turgor of mesocarp cells together with sugars. This also means that K^+^ ion accumulation, in the flesh cell vacuoles, surpasses purely nutritional requirements and participates in cell expansion mechanisms. At the whole plant level, K^+^ fluxes are involved in leaves in stomata opening and closure, and root K^+^ transport systems drive K^+^ uptake from the soil and control the emergence of root lateral primordium [103]. Given the necessity for K^+^ to be present in high concentrations in all cell types, it is necessary for the grape growers to ensure that K^+^ availibilty is sufficient to allow appropriate berry growth and functioning. On the other hand, upon the current and ongoing climate change period, it is a true challenge since an excess of K^+^ in the fruit will neutralize the organic acids, thus increasing berry pH and a decline of wine quality. Considering the importance of berry composition at harvest as a prerequisite of the production of great wines, the grape community is active on this issue. Many research focalize on the improvement of our understanding of the underlying mechanisms controlling the vine and grape responses to climatic parameters that can appear individually or in combination. QTL analyses to identify key genes involved in the control of the characters studied [169] and adequate breeding programs or the definition of specific cultural practices are required [170,171,172]. Altogether, this information will be relevant to understand grape varietal adaptation to climate changes and to assist vine growers in choosing the best genotypes.

## Figures and Tables

**Table 1:** Arabidopsis and grapevine Shaker-like voltage-gated K^+^ channels. Abbreviations: Channel subunits K_in_, K_weak_, and K_out_ form homomeric inwardly rectifying, weakly inwardly rectifying, or outwardly rectifying channels. The channel K_silent_ is silent (non-functional) when expressed alone but forms a functional heterotetrameric channel with K_in_ and K_weak_ subunits.

Gene	ID	Species	Group	Voltage Dependence	Main Tissue Localization	Function	References
AKT1	*At2g26650*	*A. thaliana*	1	K_in_	Root cortex, root hairs, guard cells	K^+^ loading by roots	[92,93]
VvK1.1	*VIT_11s0016g04750*	*V. vinifera*	1	K_in_	Root cortex and grape berry phloem	K^+^ loading by roots	[28]
VvK1.2	*VIT_04s0008g04990*	*V. vinifera*	1	K_in_	Flesh cells and perivascular cells in berries	K^+^ loading by flesh cells	[32]
AKT5	*At4g32500*	*A. thaliana*	1	K_in_	Developing seed	-	https://bar.utoronto.ca/
SPIK	*At2g25600*	*A. thaliana*	1	K_in_	Pollen	K^+^ uptake for pollen tube growth	[94]
KAT1	*At5g46240*	*A. thaliana*	2	K_in_	Guard cells	stomatal opening	[95]
KAT2	*At4g18290*	*A. thaliana*	2	K_in_	Guard cells, phloem and flower	stomatal opening	[96]
VvK2.1	*VIT_10s0003g03270*	*V. vinifera*	2	K_in_	Guard cells and seeds	stomatal opening	[89]
AKT2	*At4g22200*	*A. thaliana*	3	K_weak_	Phloem	Phloem K^+^ unloading and loading	[97]
VvK3.1	*VIT_12s0034g02240*	*V. vinifera*	3	K_weak_	Phloem and pulvinus	Phloem K^+^ unloading and loading and leaf movements	[33]
AtKC1	*At4g32650*	*A. thaliana*	4	K_silent_	Root cortex, Roots hairs, and leaf trichomes	regulatory subunit	[98,99]
VvK4.1	*VIT_04s0008g04510*	*V. vinifera*	4	K_silent_	-	-	-
GORK	*At5g37500*	*A. thaliana*	5	K_out_	Roots and root hairs and guard cells	stomatal closure—root K^+^ efflux	[100,101]
SKOR	*At3g02850*	*A. thaliana*	5	K_out_	Root stele (xylem parenchyma)	K^+^ secretion to the xylem	[102]
VvK5.1	*VIT_14s0006g00100*	*V. vinifera*	5	K_out_	Roots, phloem and flowers	K^+^ secretion to the xylem, lateral root, phloem repolarization	[103]
VvK5.2	*VIT_18s0089g01300*	*V. vinifera*	5	K_out_	-	-	-
VvK5.3	*VIT_11s0016g05810*	*V. vinifera*	5	K_out_	-	-	-
VvK5.4	*VIT_17s0000g01980*	*V. vinifera*	5	K_out_	-	-	-
